# Sustainable Magnetic Materials (from Chitosan and Municipal Biowaste) for the Removal of Diclofenac from Water

**DOI:** 10.3390/nano9081091

**Published:** 2019-07-30

**Authors:** Roberto Nisticò, Alessandra Bianco Prevot, Giuliana Magnacca, Lorenzo Canone, Sara García-Ballesteros, Antonio Arques

**Affiliations:** 1 Department of Applied Science and Technology DISAT, Polytecnic of Torino, C.so Duca degli Abruzzi 24, 10124 Torino, Italy; 2 Chemistry Department, University of Torino, 10125 Torino, Italy; 3 NIS Interdepartmental Centre, University of Torino, 10125 Torino, Italy; 4 Department of Textile and Paper Engineering, Universitat Politècnica de València, E-03801 Alcoy, Spain

**Keywords:** bio-based substances, biomasses valorization, chitosan, magnetic materials, photocatalysis, wastewater treatments

## Abstract

The photodegradation of an aqueous solution of diclofenac (DCF) has been attempted in the presence of hydrogen peroxide and organic/inorganic hybrid magnetic materials under simulated and real solar light. The hybrid magnetic materials have been prepared via coprecipitation synthesis starting from iron(II) and iron(III) inorganic salts in the presence of bioderived organic products (i.e., chitosan or bio-based substances isolated from commercially available composted urban biowastes) acting as stabilizers of the iron-containing phase. In addition to the as prepared hybrid materials, the corresponding materials obtained after a pyrolytic step at low temperature (550 °C) have been tested. The obtained results evidenced the capability of the materials to activate hydrogen peroxide at mild pH promoting DCF (photo) degradation. All the materials feature also as adsorbents since a decrease of DCF is observed also when working in the dark and in the absence of hydrogen peroxide.

## 1. Introduction

Environmental protection is considered worldwide a primary issue of sustainable development. In this context scientific research has been recently focused on two issues: The recycle and reuse of raw materials and natural resources, and the improvement of efficiency of air, waters and soils remediation techniques. As for water, the increasing anthropogenic impact on water sources is creating a dramatic depletion of the available resources and there is a rising demand for exploring integrated water use and water treatment processes seeking for a transition to more circular water management [1]. In addition to the removal of standard contaminants, a rising threaten is represented by the presence of contaminants of emerging concern, i.e., a wide class of chemicals from anthropogenic origin, hard to be removed and increasingly detected in treated and freshwaters because of their extensive use in the modern society [2,3]. Among the different water treatment approaches, advanced oxidation processes (AOPs) could represent a suitable tool, since they have given very promising results in a large number of studies concerning the removal from waters of organic compounds recalcitrant to biological treatment [4,5,6,7].

Among several AOPs, Fenton, photo-Fenton and Fenton-like processes have been attracting wide attention [8] due to their ability to generate highly reactive species. In the “classic” Fenton process, the highly reactive species, mainly hydroxyl radicals (OH), are generated by the reaction between Fe(II) ions and hydrogen peroxide. The reaction strongly depends on the pH that has to be adjusted to the optimum of 3 and subsequently it has to be neutralized, with sludge formation and increase of the overall cost of the process [9]. In the heterogeneous Fenton and photo-Fenton reactions the H_2_O_2_ is activated by iron supported in a solid matrix at acidic or even circumneutral pH; magnetite/maghemite (magnetic iron oxides) represent promising candidates for this application due to their benign nature and low-cost; furthermore, their surface can be functionalized with a wide variety of non-toxic materials containing (photo) active groups that can act both as stabilizers against the iron oxide oxidation and also as catalysts [10,11]. In addition, organic-inorganic hybrid magnetic materials feature the advantage of easy recovery from the solutions after the wastewater treatment [12].

Recently it has been proposed the use of bioderived products, with a low-cost source and an environmentally friendly nature, for the preparation of magnetic hybrid materials to be used in environmental remediation [13,14,15,16].

In the present work, two different bioderived products have been used, i.e. chitosan (an amino polysaccharide derived from very abundant chitin) and bio-based substances isolated from commercially available composted urban biowastes, to prepare the corresponding magnetic hybrid materials to be tested in the heterogeneous photo-Fenton degradation of diclofenac, a well-known analgesic and anti-inflammatory drug belonging to the contaminant of emerging concern category. In addition to the as prepared hybrid materials, also the corresponding materials obtained after a pyrolytic step at low temperature (550 °C) have been tested.

## 2. Materials and Methods 

### 2.1. Materials

Ferrous sulphate heptahydrate FeSO_4_·7H_2_O (CAS 7782-63-0, purity ≥99.5%, Sigma-Aldrich, Saint Louis, MO, USA) and ferric chloride FeCl_3_ (CAS 7705-08-0, purity ≥98.0%, Sigma-Aldrich, Saint Louis, MO, USA) were selected as precursors of the magnetite core of materials. Commercially available chitosan from crab shells (CAS 9012-76-4, medium M_w_, DD = 75–85%, Sigma-Aldrich, Saint Louis, MO, USA) and bio-based substances (BBS-GC) isolated from commercially-available composted (more than 180 days) urban biowaste from the ACEA Pinerolese Industriale S.p.A. waste treatment plant (Pinerolo, Italy) following the procedure already reported in Reference [13], were used as organic stabilizers of the magnetic nanoparticles and as active phases for pollutant removal. Diclofenac sodium salt C_14_H_11_Cl_2_NO_2_ (DCF, CAS 15307-86-5, assay ≥98.5%, Sigma-Aldrich, Saint Louis, MO, USA) was selected as a target emerging pollutant as it is a nonsteroidal anti-inflammatory drug. Other reagents used were: hydrochloric acid (CAS 7647-01-0, conc. 37 wt %, Sigma-Aldrich, Saint Louis, MO, USA), nitric acid (CAS 7697-37-2, conc. 65%, Sigma-Aldrich, Saint Louis, MO, USA), sulfuric acid (CAS 7664-93-9, conc. 85%, Montplet and Esteban S.A., Barcelona, Spain), acetic acid (CAS 64-19-7, purity ≥99.8%, Sigma-Aldrich, Saint Louis, MO, USA), ammonium acetate (CAS 631-61-8, purity ≥98%, Sigma-Aldrich, Saint Louis, MO, USA), ammonium hydroxide solution(CAS 1336-21-6, NH_3_ essay 28–30%, Merck KGaA, Darmstadt, Germany), sodium hydroxide (CAS 1310-73-2, purity ≥98.0%, Sigma-Aldrich, Saint Louis, MO, USA), potassium hydroxide (CAS 1310-58-3, assay ≥90%, Sigma-Aldrich, Saint Louis, MO, USA), potassium chloride (CAS 7447-40-7, purity ≥99.0%, Sigma-Aldrich, Saint Louis, MO, USA), hydrogen peroxide H_2_O_2_ (CAS 7722-84-1, essay 30%, Merck KGaA, Darmstadt, Germany), methanol UHPLC grade (CAS 67-56-1, essay 99.9%, Merck KGaA, Darmstadt, Germany), acetonitrile (ACN) for UHPLC grade (CAS 75-05-8, essay 99.9%, Merck KGaA, Darmstadt, Germany), 1,10-phenanthroline (*o*-phenanthroline, CAS 66-71-7, purity ≥99.0%, Sigma-Aldrich, Saint Louis, MO, USA), L-(+)-ascorbic acid (CAS 50-81-7, assay 99%, Alfa Aesar, Thermo Fisher GmbH, Karlsruhe, Germany). All aqueous solutions were prepared using ultrapure water Millipore Milli-Q^TM^. All chemicals were used without further purification.

### 2.2. Preparation of Magnetic Materials

Magnetic nanoparticles were prepared following a procedure already reported in the literature [14,15,16,17]. In detail, FeCl_3_ and FeSO_4_·7H_2_O (molar ratio Fe(III)/Fe(II) = 1.5) were dissolved in 100 mL of deionized water and heated up to 90 °C. Afterwards, two solutions were added simultaneously: (i) 10 mL of 28–30% ammonium hydroxide (to generate the basic environment necessary for the co-precipitation reaction) [18], and (ii) 50 mL of an aqueous solution containing the two selected stabilizers (i.e., either 2 wt.% BBS solution or 1 wt.% chitosan in weak acid environment). The black compounds were mechanically stirred for 30 min at 90 °C and subsequently cooled down to room temperature (RT). Magnetic precipitates were purified by freshwater washing and dried at 80 °C overnight. Carbon-coated magnetic materials were obtained by pyrolyzing the prepared magnetic materials in a quartz tube reactor LTF 12/38/500 Lenton (tube inside diameter: 38 mm, heated length: 500 mm, Lenton Furnaces and Ovens, Parsons Lane, UK) under a nitrogen atmosphere (gas flow: 250 mL min^−1^) with the following thermal program: heating ramp from RT to 550 °C (heating rate of 5 °C min^−1^), isothermal step at 550 °C for 1 h, free cooling down. Magnetic materials were coded as follows: MB (BBS-stabilized magnetite), MBP (BBS-stabilized magnetite after pyrolysis at 550 °C), MC (chitosan-stabilized magnetite), MCP (chitosan-stabilized magnetite after pyrolysis at 550 °C).

### 2.3. Characterization of Magnetic Materials

The magnetization of the obtained materials was measured by means of a LakeShore 7404 vibrating sample magnetometer (Lake Shore Cryotronics Inc., Westerville, OH, USA) registering the hysteresis loop of the samples at room temperature with a magnetic field sweeping between −20,000 and 20,000 Oe.

The morphology of the materials was investigated by electron microscopy measurements using a high-resolution transmission microscope JEOL JEM 3010 (JEOL USA, Inc., 11 Dearborn Road, Peabody, MA, USA) working at 300 kV equipped with a LaB_6_ source and a scanning microscope ZEISS EVO 50 XVP (Carl Zeiss AG, Oberkochen, Germany) equipped with LaB_6_ source and a secondary electron detector. The samples prior to the SEM investigation were sputtered with ~20 nm of a gold layer in order to avoid charging effects using a Bal-tec SCD050 sputter coater (Leica Biosystems Inc., Buffalo Grove, IL, USA).

### 2.4. Analytical Methods

Total carbon (TC), total inorganic carbon (TIC) and total organic carbon (TOC) content were determined by means of a Shimadzu TOC-VCSH analyzer (Shimadzu Corporation, Kyoto, Japan). The TC analysis was determined spectroscopically by performing complete combustion of samples at 680 °C in presence of Pt catalysts supported on alumina, and subsequently determining the amount of CO_2_ formed by means of a non-dispersive infra-red (NDIR) detector. The TIC analysis was performed following the same procedure as for TC after treatment in the presence of phosphoric acid (25%) ad air in order to decompose also the inorganic (bi) carbonates. TOC was determined as the difference between TC and TIC.

Diclofenac (DCF) concentration was determined by high-performance liquid chromatography. The apparatus was a LaChrom from Merck-Hitachi (Hitachi High-Tech Solutions Corporation, Fukuoka, Japan) equipped with an autosampler and UV diode array L-7459A detector. A reverse-phase column LiChrospher^®^ 100 RP-18 (125 mm, i.d. 4 mm, d.p. 5 μm) was used and a mixture of 1 mM sulfuric acid (30%) and acetonitrile ACN (70%) was employed as a mobile phase in a flow rate of 1.0 mL min^−1^. The retention time for DCF was 4.5 min, the total elution time was 8 min for each run; detection wavelength = 275 nm.

The quantification of DCF has been performed by sampling 1.6 mL at different times (t = 0, 1, 2, 3, 5, 10, 15, 20, 30, 45, 60, 90 min) and adding 0.3 mL of methanol (quenching the degradation reaction by reacting with the radicals) prior to performing the chromatographic analysis.

The amount of peroxide in the solution during the photocatalytic experiments was monitored by using Peroxidase Indicator Strips in the 0–100 mg L^−1^ range (Merck KGaA, Darmstadt, Germany).

The concentration of Fe(II) ions present in the suspensions was measured according to the *o*-phenanthroline standardized spectrometric procedure (ISO 6332) [7].

### 2.5. Irradiation Equipment and Photocatalytic Tests

Photocatalytic tests were performed in a 1 L cylindric photoreactor that has been described in detail elsewhere [19]. The reactor had a frit glass diffuser at the bottom to allow bubbling air into the solution, to ensure the presence of oxygen but also to stir the solution and to keep the catalyst in suspension. A flow of 3 L/h of air was kept all along the reaction time. The reactor was submerged in a thermostatic bath to keep the desired temperature. Irradiation was performed by means of a xenon lamp (Heraeus TXE 150, Heraeus Holding GmbH, Hanau, Germany), which was placed axially inside the reactor. The lamp was inside a Pyrex glass envelope that cuts off radiation below 300 nm.

Solar irradiations were performed in open glass cylindrical reactors, that were located in a sunny place. The typical solar irradiance in the UVA region was ~30 W/m^2^. For each reaction, they were loaded with 250 mL of the solution to be treated. Air was continuously bubbled with a pump to keep the catalysts in suspension.

## 3. Results and Discussion

### 3.1. Characterization Results

**Figure 1:**
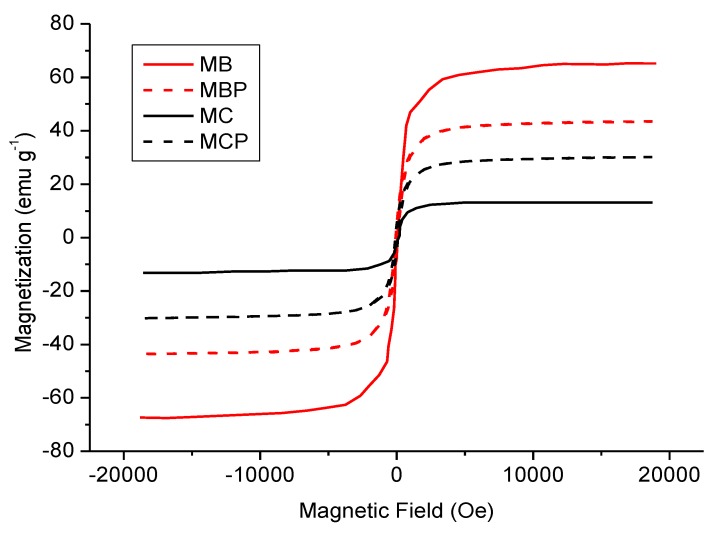
Magnetization curves related to the prepared samples. Legend: MB (BBS-stabilized magnetite), full red line, MBP (BBS-stabilized magnetite after pyrolysis at 550 °C) broken red line, MC (chitosan-stabilized magnetite), full black line, MCP (chitosan-stabilized magnetite after pyrolysis at 550 °C), broken black line.

Figure 1 reports the magnetization curves related to the prepared samples. In all cases, the samples are magnetic enough to be easily recovered from the suspension after the experiments.

**Figure 2:**
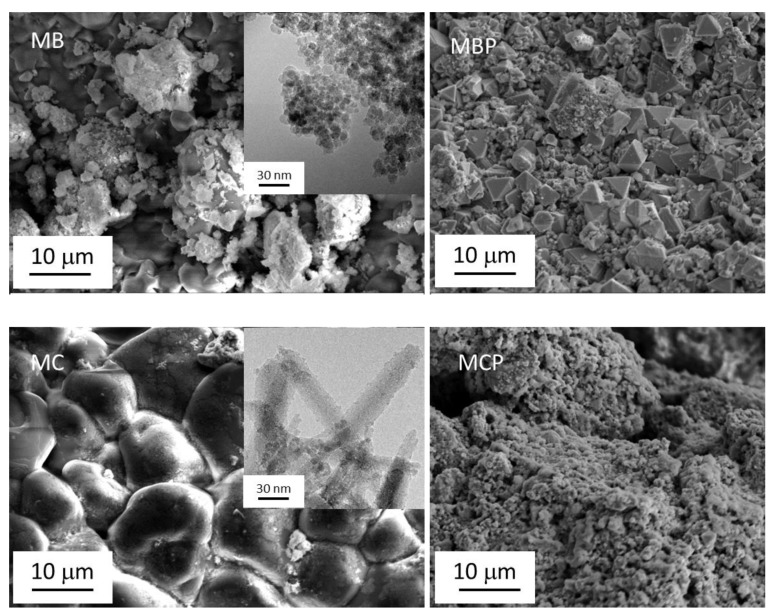
Scanning Electron Microscopy (SEM) images of MB, MC and parent pyrolyzed samples. The inset shows the Transmission Electron Microscopy (TEM) images related to the samples before pyrolysis.

The morphology of the samples was studied by means of electron microscopies SEM and TEM. The images are reported in Figure 2 MB and MC possess a very different morphology, made of aggregates of small particles in the case of MB (as better evidenced by the TEM image reported in the inset), made of large globular components in the case of MC. In the latter case, the TEM image evidenced the presence of elongated large particles of chitosan decorated with small, optically dense particles homogeneously dispersed already recognized in previous papers as magnetite/maghemite nanoparticles [16]. The pyrolysis creates different structures: octahedral particles of variable size and several defects are visible in the sample MBP, whereas the aggregates possess a flower-like structure in the case of MCP.

### 3.2. Preliminary Adsorption Experiments

#### 3.2.1. Adsorption of Diclofenac on the Nanomaterials

Prior to proceeding with the (photo) catalytic experiments, materials were dispersed in solution by means of ultrasonic bath for 10 min, in order to maximize the surface/volume ratio in the magnetic materials. Table 1 reports the amounts of Fe(II) and organic carbon released in solution by simply dispersing materials in water or after performing the ultrasonic treatment. Results evidenced that the quantity of Fe(II) and organic carbon are comparable prior and after ultrasonic treatment (thus this step does not affect the stability of the materials), confirming a limited release of Fe (lesser than ca. 0.1 mg L^−1^) and a release of organic carbon higher for the non-pyrolyzed materials with respect to the pyrolyzed ones.

**Figure 3:**
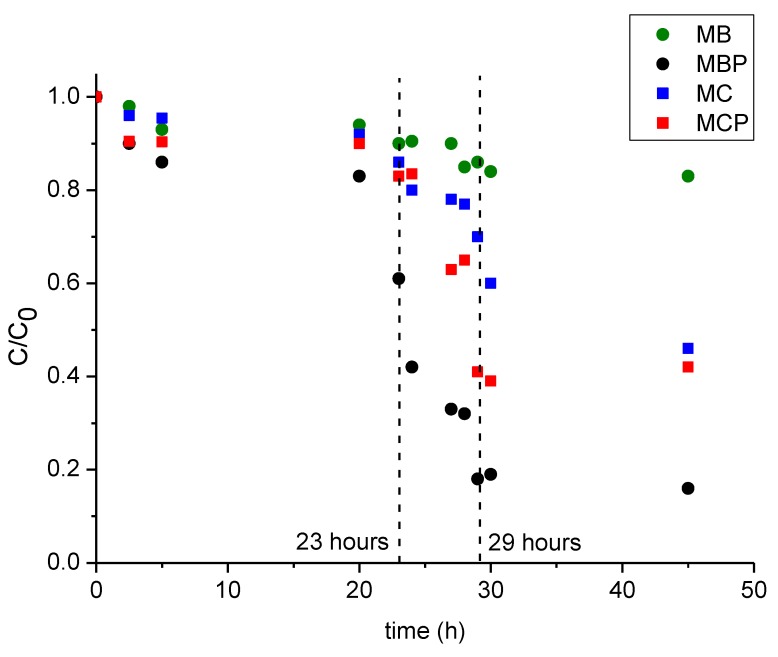
Kinetics of adsorption of DCF (C_0_ = 20 mg L^−1^) for the four magnetic materials. Data reported considering a final concentration of materials of 500 mg L^−1^ and a final time of 45 h. Legend: MB (green circles), MBP (black circles), MC (blue squares), MCP (red squares). Vertical dotted lines evidenced the two additional steps of 200 mg L^−1^ of materials at 23 and 29 h.

The aim of this study was the (photo) catalytic abatement of DCF in suspension by heterogeneous (photo) Fenton processes. However, the materials here investigated show a well-documented adsorption capacity [15,16,17], thus it must be clarified if the DCF abatement is due to either a catalytic degradation or simply adsorption. In order to clarify this point, a specific experiment was performed to quantify the extension of the adsorption phenomenon: namely, a fixed concentration of DCF (20 mg L^−1^) was mechanically shaken in presence of the materials (100 mg L^−1^) till the equilibrium of the process was reached (for up to 23 h) at RT, followed by the first addition of materials (200 mg L^−1^) until the new equilibrium was reached (up to 29 h), and a second addition of materials (200 mg L^−1^) until reaching 45 h. Subsequently, the concentration of DCF in solution was determined by HPLC analysis. Figure 3 reports the results of this set of experiments performed for the four materials. As evidenced in the graph, within the first 23 h the initial adsorption was very weak, with a more significant depletion of the DCF concentration for the two pyrolyzed materials (e.g., almost 20% of abatement for MBP). After the addition of 200 mg L^−1^ of materials, a further reduction of the DCF concentration was observable in all cases, and increasing in the order MB < MC < MCP < MBP (10% of abatement was observed for MB, whereas more than 60% of abatement was reached for MBP material) whereas a less significant change was observed after the addition of a further 200 mg L^−1^ except for MC sample which shows a final removal of 55%.

#### 3.2.2. Diclofenac (Photo) Stability

**Figure 4:**
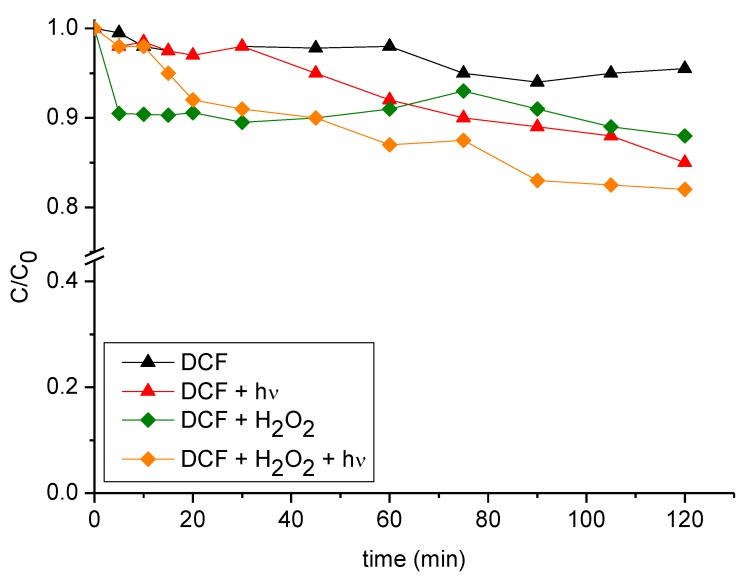
DCF concentration (C_0_ = 20 mg L^−1^) profiles without any additional substances (black triangles), under irradiation (red triangles), in presence of stoichiometric H_2_O_2_ (green diamonds), in presence of stoichiometric H_2_O_2_ and under irradiation (orange diamonds). Data reported consider a final time of 120 min.

In order to evaluate the (photo) stability of DCF under different experimental conditions, preliminary tests were performed as follows: (i) without any additional substance, (ii) by simply irradiating (DCF photolysis), (iii) in the presence of stoichiometric concentration of hydrogen peroxide H_2_O_2_ (namely, 1:33 DCF: H_2_O_2_ molar ratio), (iv) in presence of both H_2_O_2_ and irradiation. Tests (i) and (iii) have been performed in the dark. Results reported in Figure 4 evidenced that the degradation of DCF (in the dark) is below 10%, even in the presence of H_2_O_2_. On the contrary, the DCF concentration decreases of about 15% under irradiation and of ~20% by irradiating in the presence of H_2_O_2_. The enhanced DCF degradation experimentally observed in the presence of hydrogen peroxide under irradiation can be ascribed to the reaction with hydroxyl radicals (OH) generated through the H_2_O_2_ photolysis.

### 3.3. Effect of Nanomaterials on the Photodegradation of Diclofenac

**Figure 5:**
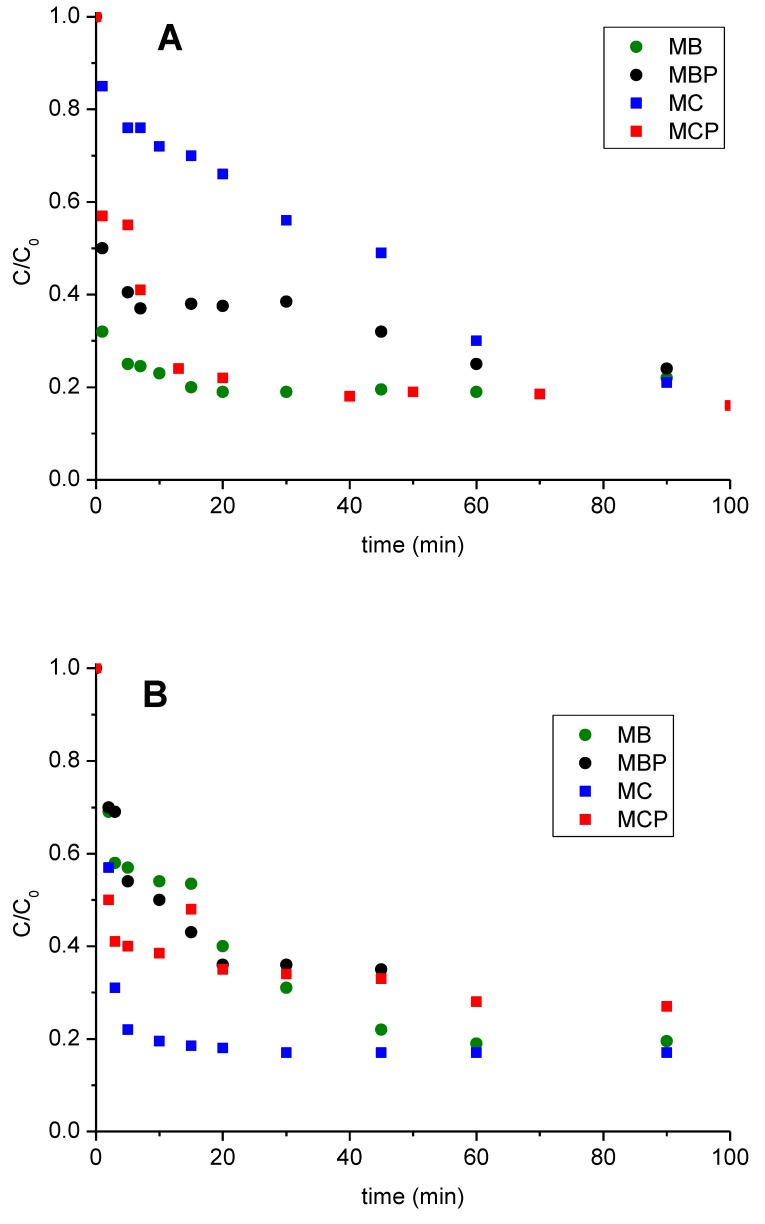

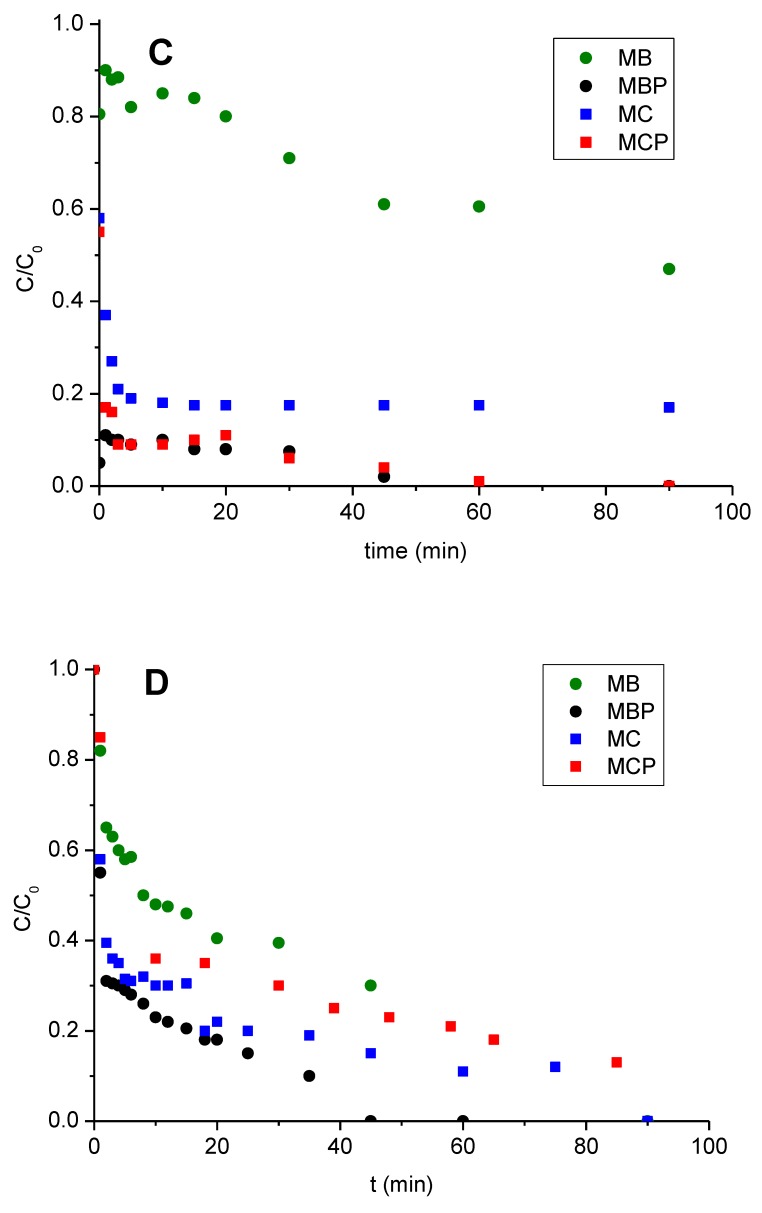
DCF concentration (C_0_ = 20 mg L^−1^) profiles measured for the four materials under study at different experimental conditions. (**A**) In the presence of only materials. (**B**) In the presence of materials and under irradiation. (**C**) In presence of materials and stoichiometric H_2_O_2_ amount. (**D**) In presence of materials, stoichiometric H_2_O_2_ amount and under irradiation. Data reported consider a final concentration of materials of 500 mg L^−1^ and a final time of 90 min. Legend: MB green circles, MBP black circles, MC blue squares, MCP red squares.

To evaluate the activity of each material, several (photo) degradation tests were carried out in the presence of DCF (10 mg L^−1^) at different experimental conditions: (i) only in the presence of materials (Figure 5A), (ii) in the presence of materials and under irradiation (Figure 5B), (iii) in the presence of materials and stoichiometric H_2_O_2_ (Figure 5C), (iv) in the presence of materials, stoichiometric H_2_O_2_ and under irradiation (Figure 5D). Tests (i) and (iii) have been performed in the dark. A remarkable reduction of the DCF concentration has been registered in the presence of MB, MBP, and MCP, probably due to a very highly pronounced sorption phenomenon, whereas this effect is less pronounced in the case of MC (Figure 5A). However, all materials reached values of residual DCF of ~20% after 90 min. Almost analogous performances were reached under irradiation (Figure 5B), even if, in this case, the best results were reached by MBP (showing the almost complete abatement of DCF after ~60 min), MC and MB (both decomposing ~80% of DCF after 90 min). The introduction of H_2_O_2_ (Figure 5C) causes an acceleration of the processes involving pyrolysed substrates MBP and MCP leaving a DCF residue close to zero after 60 min. MC kinetic profile indicates a very fast reactivity with a rapid deactivation (a plateau was reached in the first 10 min), probably due to H_2_O_2_ complete consumption. Quite surprisingly the MB profile significantly lowered, leaving a final residue of ~60%. As the presence of iron in the catalytic activity of the materials is fundamental, the trend observed can be explained considering that the release of Fe in solution is very limited for MB, rather than the other systems (see Table 1). Photo-Fenton conditions (Figure 5D) evidenced the best performances for all materials reaching in almost all cases the complete abatement of DCF, following the trend MBP > MC > MCP > MB. It must be noted that in all cases it has been registered a very pronounced reduction of DCF concentration within the first min regardless of the presence of H_2_O_2_ and/or irradiation confirming that a preliminary adsorption phenomenon of DCF at the material surface occurs.

However, according to the literature studies concerning the photo-Fenton degradation of DCF, it is possible to hypothesize that this behavior is due to a variation of the experimental parameters occurring upon the introduction of the materials in the suspension and light irradiation. In fact, the H_2_O_2_ concentration and pH monitored during the (photo) catalytic procedures revealed that in all cases the H_2_O_2_ concentration diminished from 75 mg L^−1^ to ~30 mg L^−1^ after 45 min and to 3–10 mg L^−1^ after 90 min. Interestingly, also the pH decreased from the initial value of 2.5–3.5 to ~1.5 at the end of the experiments, reasonably because of the generation of acid species (e.g., carboxylic acids) as DCF degradation intermediates. Moreover (and more important), the Fe in the solution can hydrolyze itself, thus acidifying the solution. In fact, at pH values below 3, Fe remains in solution as a ferric ion, which can be in the complexed form with water as follows:

[Fe(H_2_O)_6_]^3+^ (*aq*) + H*2*O → [Fe(H_2_O)_5_OH]^2+^ (*aq*) + H_3_O^+^ (1)

Such a decrease in pH can significantly affect the experimental conditions. In fact, as reported by Perez-Estrada and co-workers [20], DCF is an acid compound very soluble at pH > 4 (i.e., 50 g L^−1^ at 25 °C and pH 7, pKa = 4.15), whereas it is almost insoluble at pH < 4. According to this last consideration, it becomes hard to distinguish if the kinetics of abatement observed for DCF are attributable to either adsorption and/or (photo) degradation phenomena or on pH-driven precipitation induced by the addition of iron oxides, for this reason, the following experiments were carried out at controlled pH.

### 3.4. Photodegradation of Diclofenac under Controlled pH

Since a continuous control of the experimental parameters (in particular the pH) is mandatory for guaranteeing the complete solubility of DCF in the aqueous medium, further tests were performed in a proper reactor suitable for continuously controlling the parameters. In their study, Perez-Estrada and co-workers [20] evaluated that the degradation of DCF can take place in a homogeneous phase by a precipitation-redissolution-degradation process, even if DCF precipitates at low acid pH. Experiments were performed in continuous in three steps, as follows:

1. First adsorption step: where the desired amount of materials (500 mg L^−1^) was put in contact with DCF (20 mg L^−1^) for 15 min in the dark. Sampling at t = 0, 5, 10, 15 min.
2. Second Fenton step: at the previously prepared solution, was added the stoichiometric concentration of H_2_O_2_ for other 15 min in the dark. Sampling at t = 15, 20, 25, 30 min.
3. Third photo-Fenton step: at this point, the Xenon lamp has been introduced in the reactor and switch on to perform photocatalytic tests. Sampling at t = 30, 31, 32, 33, 34, 35, 37, 40, 45, 50, 60, 75, 90, 105, 120, 150 min.

During the experiments, the pH value, as well as the concentration of Fe and H_2_O_2_, was monitored at fixed times. Additionally, since the introduction of the iron oxides in the suspension favored an acidic pH, prior to performing each experiment the initial pH was corrected to circumneutrality (i.e., 6.0–7.5) by dropwise addition of a basic NaOH solution.

**Figure 6:**
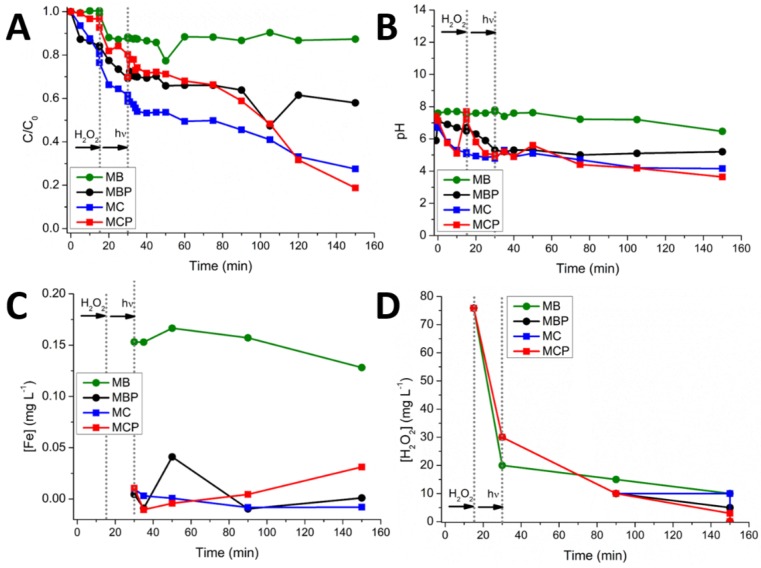
Evolution of the DCF concentration (C_0_ = 20 mg L^−1^) (**A**), pH (**B**), [Fe] (**C**), [H_2_O_2_] (**D**), during the experiments against the four different materials. Data reported consider a final concentration of materials of 500 mg L^−1^ and final time of 150 min. Legend: MB green circles, MBP black circles, MC blue squares, MCP red squares.

Figure 6 reports the DCF abatement (Figure 6A), pH values (Figure 6B), iron concentration (Figure 6C) and H_2_O_2_ concentration (Figure 6D) for the four materials. The analyses revealed that during the process the pH decreases down to 4.5. At this value, the poor quantity of Fe released in solution precipitates as hydroxide. This way, the only form of Fe available for the photo-Fenton process should be the one derived from the magnetic particles embedded in the organic matrices (acting as iron sources). The [Fe] released in solution during these processes is always very low (below 1 mg L^−1^). The consumption of H_2_O_2_ during the processes is almost linear, starting from the stoichiometric 75 mg L^−1^ to ~3–10 mg L^−1^. However, in all cases, the degradation of DCF is always partial. Figure 6A shows a first reduction due to adsorption phenomena where the best sorbent is MC (ca. 20% of the abatement). Once the H_2_O_2_ was introduced in the aqueous medium, a significant drop of the DCF amount in solution has been registered for all materials, where the best substrates seem being both MBP and MC. Lastly, the introduction of the light source favored the chitosan-derived materials (in this context, the best one is MCP), whereas the BBS-containing systems show a constant trend.

### 3.5. Experiments Performed Using Sunlight 

In order to evaluate the performance of magnetic materials using sunlight as irradiation source, specific experiments were carried out simulating the three-step experiments previously described, namely: 15 min of adsorption, followed by 15 min of Fenton reaction (simply addition of H_2_O_2_ in the dark) and subsequently sunlight irradiation (the daylight range considered was from 11.00 a.m. to 4.00 p.m.). The homogeneity of the suspensions was guaranteed by air bubbling to keep the particles in suspension [9]. Since the irradiated area is too small, it was almost impossible to calculate the time t_30_ derived considering the radiation stored. To overcome this issue, data were presented considering directly the cumulative irradiation rather than time, and consequently, the reference zero time was set once sunlight was exerted on suspensions (photo-Fenton step). Therefore, the previous 30 min (adsorption and Fenton steps) performed in the dark was considered a negative time.

**Figure 7:**
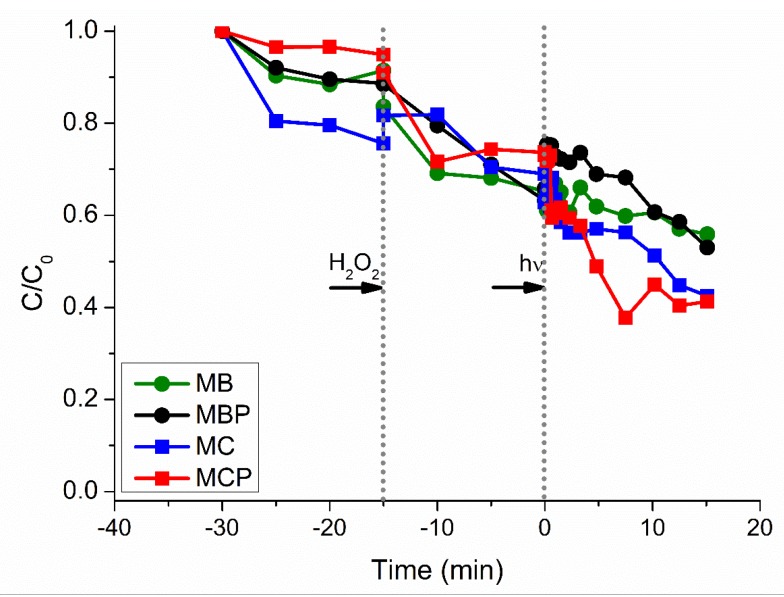
DCF concentration (C_0_ = 20 mg L^−1^) profiles against the four different materials using sunlight as the irradiation source. Data reported consider a final concentration of materials of 500 mg L^−1^ and final time of 45 min. Legend: MB green circles, MBP black circles, MC blue squares, MCP red squares. Vertical grey dotted lines evidenced the two steps, namely (i) introduction of H_2_O_2_, and (ii) beginning of the irradiation process.

Figure 7 reports the experiments performed against the four materials using the sunlight. The trends registered for pH, [Fe] and [H_2_O_2_] are analogous to the previously described experiments, with circumneutral pH, poor release of Fe (below 0.2 mg L^−1^) and peroxide consumption almost linear with a final residue of ~3–10 mg L^−1^ (data not shown for the sake of brevity). As clearly demonstrated in Figure 7, even in this case the graph shows a stepwise behavior, with the first step starting after the addition of hydrogen peroxide and the second one starting after the sunlight irradiation. Since in this particular case (i.e., sunlight source) the experimental conditions are less stable than using a Xenon lamp, the profiles of experimental curves are less linear than those obtained at laboratory scale. However, analogously to the laboratory experiments, the two chitosan-derived materials (MC and MCP) show the best performances (~40% residual DCF) with respect to the BBS-derived ones (~60%).

## 4. Conclusions

All the materials under study were prepared following an easy synthesis starting from organic refuses and were proven to be good systems for the removal of DCF from aqueous solution, behaving as adsorbents working in the dark, but also as photocatalytic materials when H_2_O_2_ in stoichiometric amount is added and/or under irradiation. This is probably due to a photo-Fenton process, running despite the mild pH values used. In addition, the magnetization measurements indicate that the materials can be easily recovered from the suspension after use for recycling.

In particular, materials prepared with chitosan (either as prepared or after pyrolysis) show better performances, especially when working under simulated solar light. Several reasons could justify this behavior and it could be hypothesized, based on the large number of NH_2_ groups present in chitosan-containing material but also maintained in the pyrolyzed chitosan-based one, that they could exhibit some action in the iron availability. For sure, this aspect is one of the most relevant, but we cannot exclude that morphological features can play some fundamental role.

Based on this premises, more work is surely needed to unveil the mechanistic aspects of the process, nevertheless, the capacity to drive photo-Fenton at mild pH conditions and under light irradiations seems to fulfill some of the principles of green chemistry deserving further research.

## Figures and Tables

**Table 1:** Fe and organic carbon released from materials before and after ultrasonic treatment.

Materials	Water		After Ultrasonic Treatment	
	[Fe] (mg L^−1^)	TOC (mg L^−1^)	[Fe] (mg L^−1^)	TOC (mg L^−1^)
MB	n.d.	12.26	0.046	14.40
MBP	0.063	8.34	0.051	8.02
MC	0.041	21.82	0.111	18.11
MCP	0.096	7.23	0.117	7.43

## References

[1] Applying the Circular Economy Lens to Water. http://circulatenews.org/2017/01/applying-the-circular-economy-lens-to-water/.

[2] Petrie B., Barden R., Kasprzyk-Hordern B. (2015). A Review on Emerging Contaminants in Wastewaters and the Environment: Current Knowledge, Understudied Areas and Recommendations for Future Monitoring. Water Res..

[3] Boxall A.B.A. (2012). New and Emerging Water Pollutants Arising from Agriculture.

[4] Bethi B., Sonawane S.H., Bhanvase B.A., Gumfekar S.P. (2016). Nanomaterials-based advanced oxidation processes for wastewater treatment: A review. Chem. Eng. Process..

[5] Andreozzi R., Caprio V., Insola A., Marotta R. (1999). Advanced Oxidation Processes (AOP) for Water Purification and Recovery. Catal. Today.

[6] Oturan M.A., Aaron J.J. (2014). Advanced oxidation processes in water/wastewater treatment: Principles and applications. A review. Crit. Rev. Env. Sci. Technol..

[7] Ghatak H.R. (2014). Advanced Oxidation Processes for the Treatment of Biorecalcitrant Organics in Wastewater. Crit. Rev. Env. Sci. Technol..

[8] Richardson S.D., Ternes T.A. (2018). Water Analysis: Emerging Contaminants and Current Issues. Anal. Chem..

[9] Minella M., Marchetti G., Laurentiis E., Malandrino M., Maurino V. (2014). Photo-Fenton oxidation of phenol with magnetite as iron source. Appl. Catal. B Environ..

[10] Nadejde C., Neamtu M., Hodoroaba V.D., Schneider R.J., Paul A., Ababei G., Panne U. (2015). Tannic acid- and natural organic matter-coated magnetite as green Fenton-like catalysts for the removal of water pollutants. J. Nanopart. Res..

[11] Nadejde C., Neamtu M., Hodoroaba V.D., Schneider R.J., Paul A., Ababei G., Panne U. (2015). Green Fenton-like magnetic nanocatalysts: Synthesis, characterization and catalytic application. Appl. Catal. B Environ..

[12] Munoz M., Pedro Z.M., Casas J.A., Rodriguez J.J. (2015). Preparation of magnetite-based catalysts and their application in heterogeneous Fenton oxidation A review. Appl. Catal. B Environ..

[13] Palma D., Prevot A., Celi L., Martin M., Fabbri D., Magnacca G., Chierotti M.R., Nisticò R. (2018). Isolation, characterization, and environmental application of bio-based materials as auxiliaries in photocatalytic processes. Catalysts.

[14] Franzoso F., Nisticò R., Cesano F., Corazzari I., Turci F., Scarano D., Prevot A., Magnacca G., Carlos L., Martire D.O. (2017). Biowaste-derived substances as a tool for obtaining magnet-sensitive materials for environmental applications in wastewater treatments. Chem. Eng. J..

[15] Nisticò R., Cesano F., Franzoso F., Magnacca G., Scarano D., Funes I.G., Carlos L., Parolo M.E. (2018). From biowaste to magnet-responsive materials for water remediation from polycyclic aromatic hydrocarbons. Chemosphere.

[16] Nisticò R., Franzoso F., Cesano F., Scarano D., Magnacca G., Parolo M.E., Carlos L. (2017). Chitosan-derived iron oxide systems for magnetically guided and efficient water purification processes from polycyclic aromatic hydrocarbons. ACS Sustain. Chem. Eng..

[17] Nisticò R., Celi L.R., Prevot A., Carlos L., Magnacca G., Zanzo E., Martin M. (2018). Sustainable magnet-responsive nanomaterials for the removal of arsenic from contaminated water. J. Hazard. Mater..

[18] (2016). Standard Methods Online Standard Methods for the Examination of Water and Wastewater. http://standardmethods.org/.

[19] Miranda M.A., Amat A.M., Arques A., Beneyto H., García A., Seguí S. (2003). Ozonisation coupled with biological degradation for wastewater treatment: A mechanistically based study. Chemosphere.

[20] Perez-Estrada L.A., Malato S., Gernjak W., Aguera A., Thurman E.M., Ferrer I., Fernandez-Alba A. (2005). Photo-Fenton degradation of Diclofenac: Identification of main intermediates and degradation pathway. Environ. Sci. Technol..

